# Psychometric properties of the Chinese version of the functional assessment of chronic illness therapy–fatigue (FACIT–F) among patients with breast cancer

**DOI:** 10.1186/s12955-023-02164-4

**Published:** 2023-08-15

**Authors:** Tingting Cai, Jialin Chen, Feixia Ni, Rui Zhu, Fulei Wu, Qingmei Huang, Tingting Zhou, Yang Yang, Changrong Yuan

**Affiliations:** 1 School of Nursing, Fudan University, 305 Fenglin Road, Shanghai, 200032 China; 2 Nursing Department, Xinhua Hospital Affiliated to Shanghai Jiao Tong University School of Medicine, Shanghai, China; 3 Department of Nursing, Department of Oncology Shanghai Medical College, Fudan University Shanghai Cancer Center, Fudan University, Shanghai, 200032 China; 4 Department of Oncology Shanghai Medical College, Fudan University, Shanghai, China

**Keywords:** Breast cancer, Fatigue, Reliability, Validity, Rasch analysis

## Abstract

**Background:**

Fatigue is the most frequent and distressing symptom affecting the physical, cognitive, and affective domains of breast cancer patients. The Functional Assessment of Chronic Illness Therapy–Fatigue (FACIT–F) has been widely used in patients with chronic diseases and has shown satisfactory reliability and validity. This study aimed to examine the psychometric properties of the FACIT–F among Chinese patients with breast cancer.

**Methods:**

Using a convenience sampling method, a cross–sectional survey (January 2020 and September 2022) was used with patients recruited from two tertiary hospitals in Shanghai, Mainland China, and a total of 597 patients completed a demographic information questionnaire, the FACIT–F and the Functional Assessment of Cancer Therapy for Breast Cancer (FACT–B). Convergent validity was estimated by calculating the Pearson correlation coefficient of the FACIT–F with the FACT–B. Measurement invariance across age was performed by examining differential item functioning (DIF) across age groups (≤ 60 and > 60 years). The internal consistency and split–half reliability were performed for reliability analysis. Unidimensionality of the scale was evaluated by the principal component analysis by Rasch analysis. Additionally, Rasch analysis was performed for item difficulty levels, and an item–person map was used.

**Results:**

No floor/ceiling effects were observed for the FACIT–F. Moderate correlations were found between FACIT–F and FACT–B (r = − 0.342, *p* < 0.01). Most items showed an absence of DIF regarding age, except for one item. In addition, the FACIT–F showed acceptable internal consistency. Principal component analysis of Rasch residuals showed that the proportion of variance explained by the FACIT–F was 53.3%, and the outfit mean square statistics for the items ranged from 0.68 to 1.90 and the infit MNSQ from 0.63 to 1.73. Additionally, an acceptable response between items and persons was found.

**Conclusions:**

The findings indicate that the Chinese version of the FACIT–F is a valid tool for the measurement of fatigue in breast cancer patients.

## Background

Breast cancer is a global public health problem. Although the improvement of early detection and the availability of effective treatments has increased the long–term survival of patients with breast cancer, they still suffer from varying degrees of distressing symptoms from cancer treatment to survivorship [1–3]. Fatigue has been considered as one of the most common adverse reactions of patients with breast cancer [4]. Cancer–related fatigue refers to the persistent fatigue caused by cancer or the relevant treatment and other disease–related factors, which affects the daily life of the patient but is not consistent with his or her activity level and is more severe and lasts longer than noncancer–related fatigue [5, 6]. Patients with fatigue often have pain, depression, insomnia, and cognitive dysfunction [7]. Significant fatigue after diagnosis may last 5 years or even longer and is closely related to decreased quality of life [8–10]. Identification of fatigue trajectories helps to understand the symptoms in this population to predict patients that will experience fatigue symptoms and improve their usual functioning and health–related quality of life. To better understand the fatigue experiences of these patients, an appropriate measure with high accuracy is needed.

A number of self–reported scales are used to measure fatigue in patients with cancer. These measures differ in the psychometric properties, the ease of administration, and the range of measurement [11]. The Functional Assessment of Chronic Illness Therapy–Fatigue (FACIT–F) are among the most recommended cancer–related fatigue outcome measures in the literature. The FACIT–F is a patient–reported outcome instrument to assess fatigue levels in addition to its impact on daily functioning, and it has been used to meet a growing demand for the precise evaluation of fatigue levels in patients with cancer [12]. In comparison to other relevant measures, the FACIT–F has been identified as one of the most suitable scales to quantify cancer–related fatigue for routine use and has shown better differentiation performance across the fatigue spectrum [11, 12]. For example, Yellen et al. [13] reported that the FACIT–F could be used as an independent, brief, unidimensional measure of fatigue. As stated by Chandran et al., although the FACIT–F is a unidimensional measure of fatigue, its items covered a wider range of fatigue, and is easy to administer and score [14]. The FACIT–F was originally derived from a 41–item survey and is now a briefer, more valid measure of fatigue [13]. With concise items, the FACIT–F still covers comprehensive aspects of fatigue, including physiology, functionality, emotionality, and sociality. Additionally, based on item response theory (IRT), the FACIT–F is more capable of guiding item selection accurately [13]. The FACIT–F has been shown to detect symptoms earlier and improve provider–patient communication, with a time frame of 7 days [15]. Although the 7–day and 4–week time frames are comparable in measuring cancer–related fatigue, the 7–day time frame was more capable of observing detailed information and is preferred [16].

Early identification and management of cancer–related fatigue during cancer treatment may help prevent a decline in quality of life through improved management of contributing factors. To better measure fatigue among breast cancer patients, brief and accurate instruments that can be used for clinical routine is needed in this population. Considering that the FACIT–F shows excellent psychometric properties in differentiating patients by fatigue level and patient–rated performance status, it is an appropriate instrument to assess fatigue in patients with cancer. Validation of the FACIT–F in in a sample of Chinese patients with breast cancer will provide a suitable and acceptable cultural fit instrument for screening and assessing fatigue in clinical practice. Therefore, this study aimed to examine the psychometric properties of the FACIT–F in a sample of Chinese patients with breast cancer according to item response theory.

## Methods

### Study populations and procedure

Using the convenience sampling method, a cross–sectional survey (January 2020 and September 2022) was used with patients recruited from two tertiary hospitals in Shanghai, Mainland China. The inclusion criteria of the patients were as follows: aged 18 or older, diagnosed with breast cancer, able to read and write independently, and volunteered to participate in the study. Patients with critical and life–threatening conditions were excluded.

All the patients were recruited and completed the survey during hospitalization. The survey was conducted with the help of trained nurse researchers to ensure standardization of data collection. Patient consent was obtained in writing before the survey, along with explanations of the purpose and process of the study. Ethical approval was approved by the Institutional Review Boards of Fudan University Cancer Hospital (no 1810192–22) and Fudan University Zhongshan Hospital (no 2020–076R).

### Measures

#### Demographic information

Demographic information collected information on age, religion, marital status, education level, lifestyle, place of residence, employment status, family income, and menstrual status.

#### Functional assessment of chronic illness therapy–fatigue (FACIT–F)

The FACIT–F is a self–reported instrument that assesses tiredness, weakness, and difficulty conducting usual activities due to fatigue over the previous 7 days [17]. The FACIT–F includes 13 items, and all items are rated on a 5–point scale, with options ranging from “not at all (0)” to “very much (4)”, and a higher score indicating higher levels of fatigue [17]. To date, the FACIT–F is available in more than 70 languages on the FACIT official website (http://www.facit.org/), and it has been widely applied in various cancer populations, such as patients with prostate cancer and colorectal cancer, and shows satisfactorily psychometric properties results [18]. Based on the PROs measures development guideline of Food and Drug Administration, we develop a Chinese version of the FACIT–F instrument for breast cancer patients with the guidance of the functional assessment of chronic illness therapy translation method. The cognitive interviews were conducted in 20 postoperative patients with breast cancer and cultural adaptation was performed. Subsequently, after a preliminary survey of psychometrics research in 246 patients with breast cancer, the final Chinese version of the FACIT–F includes 12 items, and the relevant details can be found in our previous report [19, 20].

#### Functional assessment of cancer therapy–breast (FACT–B)

The FACT–B is a frequently used instrument designed to assess quality of life in breast cancer patients [21, 22]. It includes 36 items and five subscales, encompassing physical, social, emotional, and functional well–being domains, along with a breast cancer–specific subscale. Item responses are scored based on a 5–point Likert scale, with higher scores representing a high quality of life [21]. The Chinese version of the scale has been widely validated in breast cancer patients [23]. Cronbach’s α coefficient of the FACT–B ranged from 0.87 to 0.91 in this study.

### Statistical analysis

All the analyses were performed in IBM SPSS (version 21.0), Mplus (version 7.0), and Winsteps software (version 3.75.0). Statistical significance was set at *p* < 0.05.

The descriptive statistics were conducted in which categorical data were presented as frequency and percentage and continuous data were presented as mean (standard deviation). Floor and ceiling effects were examined to assess the coverage of the FACIT–F. There was evidence of floor and ceiling effects when over 15% of respondents reported the lowest or highest score, respectively [24]. Convergent validity was performed to examine associations between the FACIT–F and the FACT–B using Pearson correlation coefficients, in which values ≥ 0.50 were considered strong correlations, 0.30–0.49 were considered moderate correlations, and ≤ 0.29 were considered weak correlations [25]. Moderate to high correlations were expected to be identified in the two measures. Differential item functioning (DIF) indicates whether respondents from various groups perform differently on the instrument even though they have the same degree of the studied trait [26]. If the absolute value of the DIF Contrast is greater than 1.0 logits, the item is considered to have a DIF [26]. We tested whether measurement bias existed across age groups (≤ 60 and > 60 years) in the scale. Regarding reliability, Cronbach’s α coefficient and split–half reliability for the scale were calculated, and a value of 0.70 or higher was deemed to indicate acceptable reliability [27, 28].

A Rasch analysis was used in this study to estimate the item or ability parameters of the FACIT–F based on item response theory [29, 30]. The Rasch model assumes that the scale based on item response theory is unidimensional, and principal components analysis (PCA) of the residuals can be used to test the unidimensionality, with the proportion of variance explained by a measure of more than 20% indicating that the data fit a unidimensional model [31]. Infit and outfit MNSQ were used to indicate the fitness of items, and values less than 2.0 were considered fitting to the model [32]. A bubble chart was used to visualize the measurement value of the model fit, and item difficulty levels were calculated during the calibration process in logits, with values in the range of − 3 to 3 being acceptable [33]. In addition, an item–person map distribution was examined to identify whether items and responders were in hierarchical order. The ideal relative difficulty and relative ability should be observed on the same interval continuum of logits [34].

## Results

### Demographic characteristics

Six hundred forty–seven patients were eligible to participate; of these, 597 (92.3%) returned valid questionnaires. The participants were an average of 48.12 years of age (SD = 10.03 years), and all of them were female. The majority of the participants reported no religious beliefs (91.3%), married (94.3%), with a senior high school education or more (43.4%), living with their families (94.3%), in a village or countryside (45.0%), were unemployed (44.6%), had low income (51.3%), and were postmenopausal (52.4%) (Table 1).

**Table 1:** Demographic characteristics of the study sample (N = 597)

Variables	n (%)
Age (years)	
18–40	126 (21.1)
41–59	390 (65.3)
≥ 60	81 (13.6)
Religion	
Yes	52 (8.7)
No	545 (91.3)
Marital status	
Married	563 (94.3)
Single/divorced/widowed	34 (5.7)
Education level	
Primary school or less	152 (25.4)
Junior high school	186 (31.2)
Senior high school or more	259 (43.4)
Lifestyle	
Living with family	563 (94.3)
Living alone	18 (3.0)
Others	16 (2.7)
Residence	
City	185 (31.0)
County	143 (24.0)
Village or countryside	269 (45.0)
Employment status	
Employed	217 (26.3)
Retired	114 (19.1)
Unemployed	266 (44.6)
Income (monthly)	
< 3000¥	306 (51.3)
≥ 3000¥	291(48.7)
Menstrual status	
Premenopausal	284 (47.6)
Postmenopausal	313 (52.4)

### Descriptive statistics

No floor/ceiling effects were observed for the FACIT–F since participants who scored the lowest score and highest accounted for 0.2% and 0.8% of the sample, respectively, and the proportion was less than 15%.

### Concurrent validity

The correlation between the FACIT–F and FACT–B was analyzed using Pearson correlation to examine the concurrent validity of the FACIT–F. The FACIT–F score was significantly correlated with the FACT–B (r = − 0.342, *p* < 0.01), supporting convergent validity.

### Measurement invariance

As shown in Table 2, all items exhibited no evidence of DIF exception for one item, with only a DIF contrast of 1.58, providing evidence in support of overall unbiased results for the scale.

**Table 2:** Differential item functioning (DIF) by age (N = 597)

Item	DIF (age < 60)	DIF (age ≥ 60)	DIF Contrast
Item 1	–0.10	–0.04	–0.06
Item 2	–0.13	0.01	–0.14
Item 3	0.17	0.09	0.09
Item 4	–0.07	0.24	–0.31
Item 5	–0.04	0.25	–0.29
Item 6	–1.50	–1.24	–0.26
Item 7	–1.38	–1.22	–0.16
Item 8	–0.13	–0.71	1.58
Item 9	1.82	1.44	0.38
Item 10	0.27	0.31	–0.04
Item 11	1.03	0.87	0.16
Item 12	0.27	–0.01	0.28

### Rasch analysis

The results of PCA supported the unidimensionality of the FACIT–F, and the proportion of variance explained by the FACIT–F was 53.3%, providing evidence of a one–factor structure. The results also indicated acceptable reliability, with an IRI of 0.99 and a PRI of 0.86, higher than the cutoff value of 0.80 (Table 3).

**Table 3:** Dimensionality analysis of the FACIT–F (N = 597)

	Empirical		Modeled	
	**Eigen**	**%**	**%**	
Total raw variance	25.7	100.0	100.0	
Raw variance explained by measures	13.7	53.3	54.7	
Raw variance explained by persons	6.9	26.7	27.4	
Raw variance explained by items	6.8	26.6	27.3	
Raw unexplained variance (total)	12,0	46.7	45.3	

The outfit MNSQ for the items ranged from 0.64 to 1.90, and the infit MNSQ ranged from 0.63 to 1.73, which were within the range of − 3 to 3. The results of the Rasch standard error calculation of the FACIT–F are shown in Table 4, suggesting that the scale was reliable. The difficulty levels of all items ranged from − 1.27 to 1.47, supporting that the items were capable of distinguishing fatigue levels across a wide range.

**Table 4:** Item parameter estimation results of the FACIT–F (N = 597)

Item	Difficulty	S.E.	Infit MNSQ	Outfit MNSQ
Item 1	–0.04	0.06	0.71	0.73
Item 2	0.01	0.06	0.69	0.69
Item 3	0.09	0.06	0.70	0.79
Item 4	0.20	0.06	0.65	0.68
Item 5	0.22	0.06	0.63	0.64
Item 6	–1.27	0.05	1.36	1.48
Item 7	–1.22	0.05	1.53	1.59
Item 8	–0.65	0.05	1.73	1.90
Item 9	1.47	0.07	1.24	1.10
Item 10	0.31	0.06	1.12	1.12
Item 11	0.87	0.06	0.84	0.77
Item 12	0.02	0.06	0.92	0.73

**Fig. 1:**
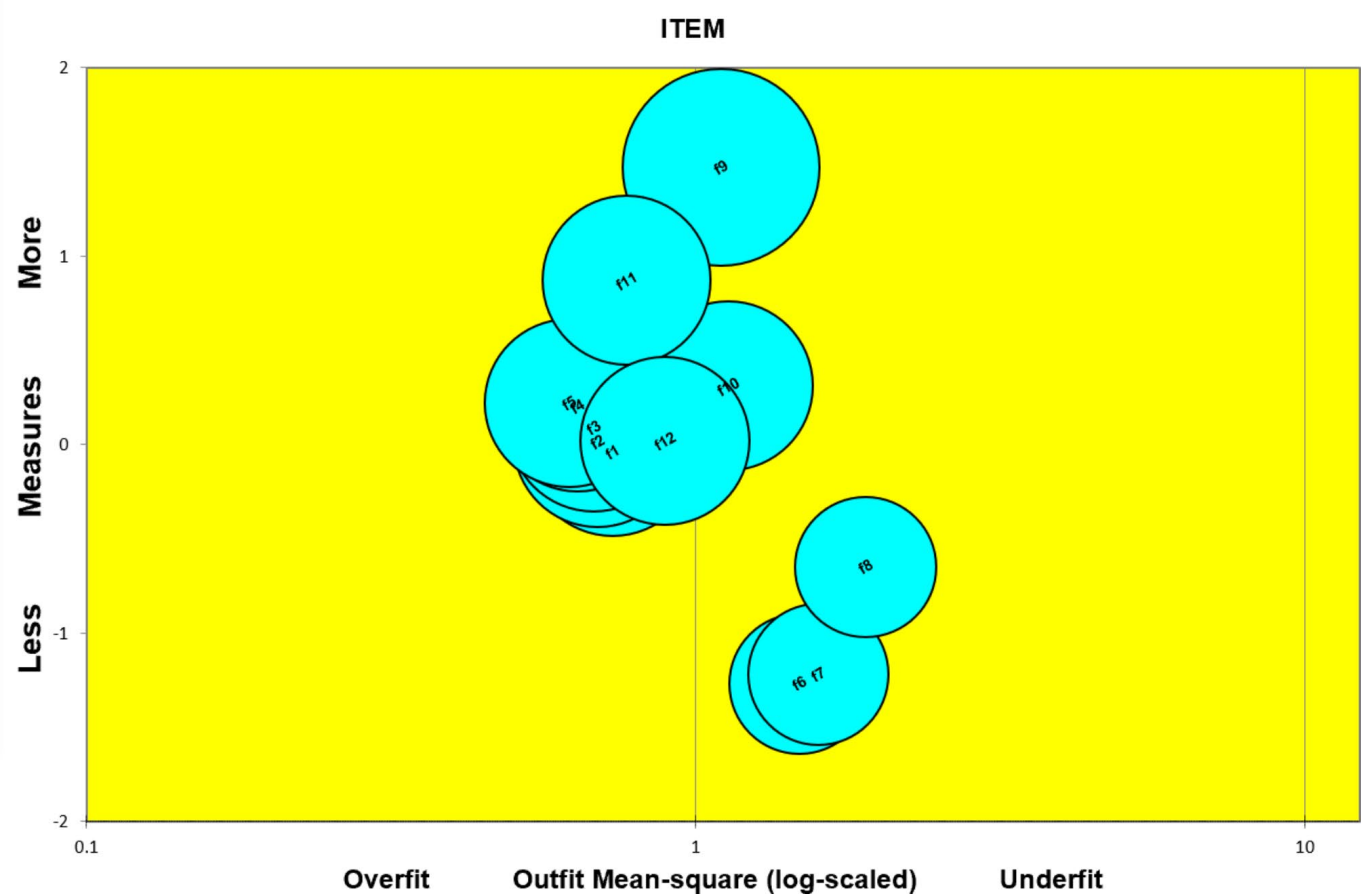
Item bubble chart of the FACIT–F

In the bubble chart, the farther to the left the item is represents overfit, the farther to the right is underfit, and items with partial overlap indicates similar difficulty. The item bubble chart of FACIT–F showed that the left was overfit while the item to the right was underfit, and most items should fall near the middle of the scale, which was in line with the hypothesis that high–quality items should be close to the middle line. Therefore, most items did not show a significant Rasch standard error. Items with partial overlap indicated similar difficulty in Fig. 1, and the standard error of the items was 0.05–0.07, indicating negligible measurement error.

**Fig. 2:**
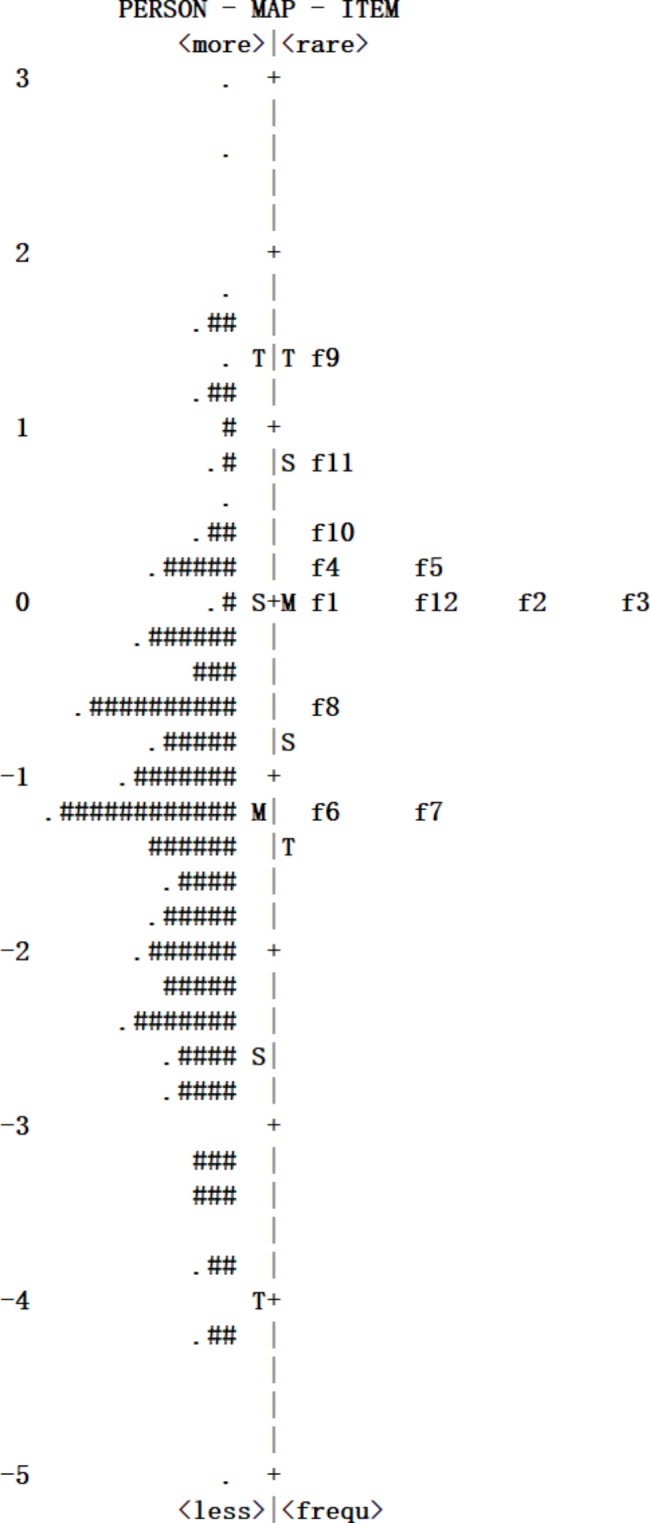
Item–person map of FACIT–F

The relationship between the item difficulty of the FACIT–F in the current sample is shown in Fig. 2, in which the left part of the figure displayed the distribution of a person’s ability levels, the leftmost number represented the logit unit of the ability of the person and item difficulty, and the right part showed the difficulty distribution of the items. From the top down, the ability level of the person decreased gradually, and the difficulty of the items decreased similarly. The most difficult item for the participants was Fatigue09 (“I am too tired to eat”) with a 1.47 logit (SE = 0.07), and the easiest item was Fatigue06 (“I have energy”) with a − 1.27 logit (SE = 0.05). By comparing the distribution of person’s ability and item difficulty, the distribution of respondent’s ability was wide, in the range of (–5–+2), but more concentrated in the range of (–3–0), and the distribution of item difficulty was narrower than the ability distribution, which was concentrated in the range (–1.5–+1.5). Thus, the results indicated that FACIT–F can well distinguish the patients with fatigue levels in the range of (–1.5–+1.5).

### Reliability

The Cronbach’s α coefficient of the FACIT–F was 0.901, and the split–half reliability was 0.922, indicating satisfactory reliability.

## Discussion

To the best of our knowledge, this is the first psychometric study of the FACIT–F specifically validated in a Chinese breast cancer population. The current results have shown that the FACIT–F had acceptable levels of reliability and validity.

Regarding convergent validity, moderate correlations between FACIT–F and FACT–B were identified, and the negative correlations revealed that the FACIT–F was correlated with measures of quality of life, and the results were similar to the original validation of the instrument [35]. FACT–B is a widely used measure previously shown to be reliable in breast cancer patients. Therefore, our results suggested that higher scores on the FACIT–F were associated with lower scores on the FACT–B, which was consistent with the commonly recognized conclusion that patients with significant fatigue symptoms were more likely to report low quality of life. Wang et al. [36] examined the psychometric properties of the Chinese version of the FACIT–F in patients receiving maintenance dialysis and found that fatigue scores were associated with several quality of life domains, including fatigue, depression, anxiety and sleep quality. Ishikawa et al. [37] validated the Portuguese version of the FACT–F in a sample of Brazilian cancer patients and compared the FACT–F to the MOS 36–Item Short–Form Health Survey (SF–36) to establish its convergent validity. The Pearson correlation results indicated acceptable correlations between the FACT–F and SF–36 scores, ranging from 0.31 to 0.76, which was in line with our results. Similarly, the FACIT–Fatigue was reported to have moderate to high correlations with the SF–36 in patients with systemic lupus erythematosus [38]. Eek et al. [39] also found strong correlations between the FACT–F and the fatigue scale in the EORTC QLQ–C30 in patients with chronic lymphocytic leukemia, with all Pearson’s r ≥ 0.5. A previous study confirmed a negative association between the FACIT–F and quality of life domains in a population with breast cancer [39].

Measurement invariance of the FACIT–F was established across age groups. The results supported that most items showed an absence of DIF with regard to age. However, one out of 12 items in the FACIT–F had DIF. The possible DIF might be due to translational differences. Sample representative might also have an impact on the psychometric properties since most of the included participants were middle–aged women with breast cancer. Since elderly patients accounted for only a small proportion of the sample, selection bias might have caused the differences. Additionally, exhausted patients were more likely to refuse to participate in the investigation, which may have an impact on the results. Overall, the establishment of measurement equivalence was in line with previous studies, showing that the FACIT–F has the same perception between populations and across ages. Kwakkenbos et al. [12] also assessed the cross–language measurement equivalence of the FACIT–F in three language versions in systemic sclerosis patients and showed that the magnitude of the DIF was negligible. Although some items had statistically significant DIF, no substantive differences were found for the FACIT–F across different language versions of the FACIT–F, which was in line with our findings.

The FACIT–F has been proposed as a unidimensional scale with the range of factor loadings being acceptable. Our results of PCA confirmed the unidimensionality of the scale, which was in line with reports in the Arabic cancer population, supporting that the items in the FACIT–F assessed a unidimensional attribute of fatigue [40]. Additionally, our results were consistent with findings reported by Kwakkenbos et al. [12], in which the FACIT–F showed a unidimensional construct in the English, French and Dutch versions of the scale.

In Rasch analyses, we found that the scale had acceptable difficulty levels, ranging from − 1.27 to 1.47, and fell into the scope of (–3, 3). In the item bubble chart, one bubble represented one item, and the size of the bubble represented the size of the measurement error. The bubble chart also demonstrated that the standard error of the items was negligible. In addition, the acceptable reliability of the scale (IRI = 0.99, PRI = 0.86) was found in the Rasch analysis. Considering this, our results were consistent with the hypothesis of IRT analysis and showed that the FACIT–F had good coverage of the fatigue domain and acceptable term difficulty distribution.

In this study, acceptable Cronbach’s α coefficients were found for the FACIT–F, reaching acceptable standards of 0.70, which compared favorably with the findings of the original English version reported by Yellen et al. [13] (α = 0.93). There were no floor/ceiling effects, showing that the items of the scale covered a wide range of fatigue levels, and the results were consistent with Maqbali et al. [40]. Test–retest reliability has not been examined in this study. Test–retest reliability of the FACIT–F has been tested in a population of patients treated for cancers of the head and neck [35]. The results confirmed that the FACIT–F demonstrated a slight advantage over the Modified Brief Fatigue Inventory with respect to test–retest reliability. The Test–retest reliability of the FACIT–F in breast cancer patients could be tested in the future.

### Limitation

Several limitations should be addressed in this study. First, we used a cross–sectional study design, and the test–retest reliability of the FACIT–F had not been assessed. Second, we did not control for demographic and clinical variables of patients with breast cancer, which might be associated with fatigue levels and could affect the results. Third, only female patients were included in this study. Thus, the measurement invariance of the FACIT–F across sexes could be tested in the future. Despite these limitations, our study provided evidence that the Chinese version of the FACIT–F was a valid tool for assessing fatigue in breast cancer patients.

## Conclusions

This study found evidence for acceptable validity and internal consistency of the FACIT–F in a sample of Chinese patients with breast cancer. It has been established that the FACIT–F can be applied to patients with breast cancer by researchers or clinicians for the measurement of fatigue in patients with breast cancer.

## Data Availability

All data presented in this paper are available from the corresponding authors on reasonable request.

## References

[1] Cai T, Zhou T, Huang Q, Wu F, Ni F, Yuan C (2023). Cancer–related symptoms among young and middle–aged women undergoing chemotherapy for breast cancer: application of latent class analysis and network analysis[J]. Eur J Oncol Nurs.

[2] Duffy S, Vulkan D, Cuckle H, Parmar D, Sheikh S, Smith R (2020). Annual mammographic screening to reduce breast cancer mortality in women from age 40 years: long–term follow–up of the UK Age RCT. Health Technol Asses.

[3] Lovelace DL, McDaniel LR, Golden D (2019). Long-term effects of breast cancer surgery, treatment, and survivor care. J Midwifery Heal.

[4] Berger AM, Gerber LH, Mayer DK (2012). Cancer–related fatigue: implications for breast cancer survivors. Cancer.

[5] Ma Y, He B, Jiang M, Yang Y, Wang C, Huang C (2020). Prevalence and risk factors of cancer–related fatigue: a systematic review and meta–analysis. Int J Nurs Stud.

[6] Abrahams HJG, Gielissen MFM, Schmits IC, Verhagen CAHHVM, Rovers MM, Knoop H (2016). Risk factors, prevalence, and course of severe fatigue after breast cancer treatment: a meta–analysis involving 12327 breast cancer survivors. Ann Oncol.

[7] Ruiz–Casado A, Álvarez–Bustos A, de Pedro CG, Méndez–Otero M, Romero–Elías M (2021). Cancer–related fatigue in breast cancer survivors: a review. Clin Breast Cancer.

[8] Minton O, Stone P (2008). How common is fatigue in disease–free breast cancer survivors? A systematic review of the literature. Breast Cancer Res Tr.

[9] Bower JE, Ganz PA, Desmond KA, Bernaards C, Rowland JH, Meyerowitz BE (2006). Fatigue in long-term breast carcinoma survivors: a longitudinal investigation. Cancer.

[10] Schmidt ME, Chang–Claude J, Vrieling A, Heinz J, Flesch–Janys D, Steindorf K (2012). Fatigue and quality of life in breast cancer survivors: temporal courses and long–term pattern. J Cancer Surviv.

[11] Minton O, Stone P (2009). A systematic review of the scales used for the measurement of cancer–related fatigue (CRF). Ann Oncol.

[12] Kwakkenbos L, Willems LM, Baron M, Hudson M, Cella D, van den Ende CHM (2014). The comparability of English, French and Dutch scores on the Functional Assessment of Chronic Illness therapy–fatigue (FACIT–F): an assessment of differential item functioning in patients with systemic sclerosis. PLoS ONE.

[13] Yellen SB, Cella DF, Webster K, Blendowski C, Kaplan E (1997). Measuring fatigue and other anemia–related symptoms with the Functional Assessment of Cancer Therapy (FACT) measurement system. J Pain Symptom Manag.

[14] Chandran V, Bhella S, Schentag C, Gladman DD (2007). Functional assessment of chronic illness therapy–fatigue scale is valid in patients with psoriatic arthritis. Ann Rheum Dis.

[15] Howell D, Molloy S, Wilkinson K, Green E, Orchard K, Wang K (2015). Patient–reported outcomes in routine cancer clinical practice: a scoping review of use, impact on health outcomes, and implementation factors. Ann Oncol.

[16] Lai JS, Cook K, Stone A, Beaumont J, Cella D (2009). Classical test theory and item response theory/Rasch model to assess differences between patient–reported fatigue using 7–day and 4–week recall periods. J Clin Epidemiol.

[17] Cella D, Wilson H, Shalhoub H, Revicki DA, Cappelleri JC, Bushmakin AG (2019). Content validity and psychometric evaluation of Functional Assessment of Chronic Illness therapy–fatigue in patients with psoriatic arthritis. J Patient Rep Outcomes.

[18] Demmelmaier I, Brooke HL, Henriksson A, Mazzoni AS, Bjørke ACH, Igelström H (2021). Does exercise intensity matter for fatigue during (neo-) adjuvant cancer treatment? The Phys‐Can randomized clinical trial. Scand J Med Sci Spor.

[19] Wu W. The Development of a Phase–specific Patient–reported Outcomes Measurement System–Breast Cancer, senior thesis of Naval Military Medical University, 2019.

[20] Huang Q, Geng Z, Fang Q, Stinson J, Yuan C (2021). Identification of distinct profiles of Cancer–related fatigue and Associated Risk factors for breast Cancer patients undergoing chemotherapy: a latent class analysis. Cancer Nurs.

[21] Brady MJ, Cella DF, Mo F, Bonomi AE, Tulsky DS, Lloyd SR (1997). Reliability and validity of the Functional Assessment of Cancer therapy–breast quality–of–life instrument. J Clin Oncol.

[22] Yoo HJ, Ahn SH, Eremenco S, Kim H, Kim WK, Kim SB (2005). Korean translation and validation of the functional assessment of cancer therapy–breast (FACT–B) scale version 4. Qual Life Res.

[23] Ng R, Lee CF, Wong NS, Luo N, Yap YS, Lo SK (2012). Measurement properties of the English and chinese versions of the functional Assessment of cancer therapy—breast (FACT–B) in asian breast cancer patients. Breast Cancer Res Tr.

[24] Katajapuu N, Laimi K, Heinonen A, Saltychev M (2019). Floor and ceiling effects of the World Health Organization Disability Assessment schedule 2.0 among patients with chronic musculoskeletal pain. Int J Rehabil Res.

[25] Portoghese I, Lasio M, Conti R, Mascia ML, Hitchcott P, Agus M (2020). Cognitive flexibility inventory: factor structure, invariance, reliability, convergent, and discriminant validity among italian university students. Psych J.

[26] Hung M, Voss MW, Bounsanga J, Gu Y, Granger EK, Tashjian RZ (2018). Psychometrics of the patient–reported outcomes Measurement Information System physical function instrument administered by computerized adaptive testing and the disabilities of arm, shoulder and hand in the orthopedic elbow patient population. J Shoulder Elb Surg.

[27] He J, Liu Y, Cheng C, Fang S, Wang X, Yao S (2021). Psychometric Properties of the Chinese Version of the 10–Item ruminative response scale among undergraduates and depressive patients. Front Psychiatry.

[28] Kes D, Gökdoğan F (2020). Reliability and validity of a turkish version of the hypertension self–care profile. J Vasc Nurs.

[29] Müller M, Haenni Hoti A (2020). Item analysis of the KIDSCREEN–10 using rasch modelling. Health Qual Life Out.

[30] Papini N, Kang M, Ryu S, Griese E, Wingert T, Herrmann S (2021). Rasch calibration of the 25–item Connor–Davidson resilience scale. J Health Psychol.

[31] Chen W, Liang Y, Yin X, Zhou X, Gao R (2021). The factor structure and Rasch Analysis of the fear of COVID–19 scale (FCV–19S) among chinese students. Front Psychol.

[32] Wu MH, Chong KS, Huey NG, Ou HT, Lin CY (2021). Quality of life with pregnancy outcomes: further evaluating item properties for refined Taiwan’s FertiQoL. J Formos Med Assoc.

[33] Hung M, Voss MW, Bounsanga J, Crum AB, Tyser AR (2017). Examination of the PROMIS upper extremity item bank. J Hand Ther.

[34] Cleanthous S, Barbic SP, Smith S, Regnault A (2019). Psychometric performance of the PROMIS® depression item bank: a comparison of the 28–and 51–item versions using Rasch measurement theory. J Patient Rep Outcomes.

[35] Eden MM, Kunkel K (2016). Psychometric properties of the modified brief fatigue inventory and FACIT–Fatigue in individuals with cancer of the head and neck. Rehabil Oncol.

[36] Wang SY, Zang XY, Liu JD, Gao M, Cheng M, Zhao Y (2015). Psychometric properties of the Functional Assessment of Chronic Illness therapy–fatigue (FACIT–Fatigue) in chinese patients receiving maintenance dialysis. J Pain Symptom Manag.

[37] Ishikawa NM, Thuler LCS, Giglio AG, Baldotto CSDR, de Andrade CJC, Derchain SFM (2010). Validation of the portuguese version of functional assessment of cancer therapy–fatigue (FACT–F) in brazilian cancer patients. Support Care Cancer.

[38] Lai JS, Beaumont JL, Ogale S, Brunetta P, Cella D (2011). Validation of the functional assessment of chronic illness therapy–fatigue scale in patients with moderately to severely active systemic lupus erythematosus, participating in a clinical trial. J Rheumatol.

[39] Eek D, Ivanescu C, Corredoira L, Meyers O, Cella D (2021). Content validity and psychometric evaluation of the Functional Assessment of Chronic Illness therapy–fatigue scale in patients with chronic lymphocytic leukemia. J Patient Rep Outcomes.

[40] Maqbali M, Hughes C, Gracey J, Rankin J, Hacker E, Dunwoody L (2020). Psychometric properties of the arabic version of the Functional Assessment of Chronic Illnesses therapy–fatigue in arabic cancer patients. J Pain Symptom Manag.

